# Gastric Antral Web as an Uncommon Cause of Gastric Outlet Obstruction in an Adult Female Patient: A Case Report of a Rare Medical Condition and a Review of the Literature

**DOI:** 10.7759/cureus.115601

**Published:** 2026-09-01

**Authors:** Jad Ozeir, Ghassan El Ouzeir, Oussama Ozeir, Mustapha El Haj Hassan, Ahmad Dalla

**Affiliations:** 1 Department of Ophthalmology, Lomonosov Moscow State University, Moscow, RUS; 2 Department of Family Medicine, Jašiūnų šeimos klinika, Uždaroji akcinė bendrovė, Jašiūnai, LTU; 3 Department of General Surgery, Lebanese University, Faculty of Medical Sciences, Hadath, LBN

**Keywords:** antral web, endoscopy, gastric antral web, gastric outlet obstruction, gastrointestinal obstruction, gastrojejunostomy, subtotal gastrectomy

## Abstract

Gastric antral web (GAW) is a rare congenital or acquired mucosal anomaly characterized by a membranous septum within the gastric antrum and represents an uncommon cause of gastric outlet obstruction (GOO), particularly in adults. Due to its nonspecific clinical presentation, it is often underdiagnosed and may lead to delayed management. We report the case of a previously healthy adult female patient who presented with progressive epigastric pain and early postprandial fullness. Physical examination and laboratory findings were unremarkable. Upper gastrointestinal endoscopy revealed a membranous structure in the antral region causing significant luminal narrowing, consistent with a GAW. A contrast study demonstrated delayed gastric emptying with passage of contrast through a narrowed aperture. Given the severity of the obstruction, the patient underwent subtotal gastrectomy with gastrojejunostomy. The procedure was uneventful, and postoperative imaging confirmed normal contrast passage without leakage or obstruction. The patient was discharged on postoperative day 2 and remained asymptomatic at the two-month follow-up. GAW remains a rare etiology of GOO, with clinical manifestations varying according to the degree of luminal narrowing. Diagnosis relies primarily on endoscopy and imaging, though a high index of suspicion is essential. Management options range from conservative measures to endoscopic and surgical interventions, with surgery remaining the definitive treatment in cases of significant obstruction. GAW should therefore be considered in the differential diagnosis of GOO in adults, as early recognition and appropriate management are crucial to prevent complications and ensure favorable outcomes, given the rarity of this condition.

## Introduction

Gastric antral web (GAW) is a congenital mucosal anomaly, typically a thin, membranous septum oriented perpendicular to the gastric antrum [1], that represents an uncommon but clinically significant cause of gastric outlet obstruction (GOO) [2,3]. These structures, which range from fenestrated diaphragms to mucosal crescents located 1-2 cm proximal to the pylorus, produce symptoms that correlate with the size of the central aperture; obstruction rarely occurs if the diameter exceeds 1 cm [2-4]. Although generally considered rare, the true prevalence remains unknown, and while historically only 44 cases had been reported by 1977, increased awareness and improved diagnostics have led to more frequent recognition [3,5]. The condition shows a male predominance (approximately 67%) [2]. Radiographically, GAW appears as a sharp, transverse band-like defect on barium studies, with a characteristic "jet phenomenon" through a narrow orifice [1,6,7]; however, diagnosis requires a high index of suspicion, as webs may be mistaken for the pylorus or other entities [6,7]. Endoscopy remains the definitive method for diagnosis [2]. Management ranges from conservative observation in partially obstructing cases to surgical resection with pyloroplasty (the gold standard for symptomatic patients), though endoscopic techniques are increasingly feasible [2,8]. Understanding this entity is essential for timely recognition and optimal outcomes in affected children and adults [1,2,5,8]. Published articles on this condition highlight not only its rarity but also the importance of early diagnosis for better outcomes and quality of life, given the limited knowledge among physicians.

## Case presentation

A previously healthy adult female patient presented with a history of intermittent epigastric pain and early postprandial fullness. The symptoms had been progressively worsening over time and were suggestive of upper gastrointestinal obstruction. The symptoms were intermittent and varied with the type of food consumed, and their timing and severity were inconsistent. She denied nausea, vomiting, weight loss, or prior gastrointestinal disease. Her medical and surgical history was unremarkable. On physical examination, the patient was hemodynamically stable, with no abdominal tenderness, distension, or palpable masses. Laboratory investigations were within normal limits.

Given the persistence of symptoms, an upper gastrointestinal endoscopy was performed. Endoscopic evaluation revealed normal esophageal mucosa without evidence of inflammation or structural abnormalities. The gastric body appeared normal; however, a membranous structure was identified in the antral region, causing significant luminal narrowing consistent with a GAW (Figure 1). The lesion was associated with functional GOO and intermittent pyloric spasm, correlating with the patient’s clinical presentation.

**Figure 1:**
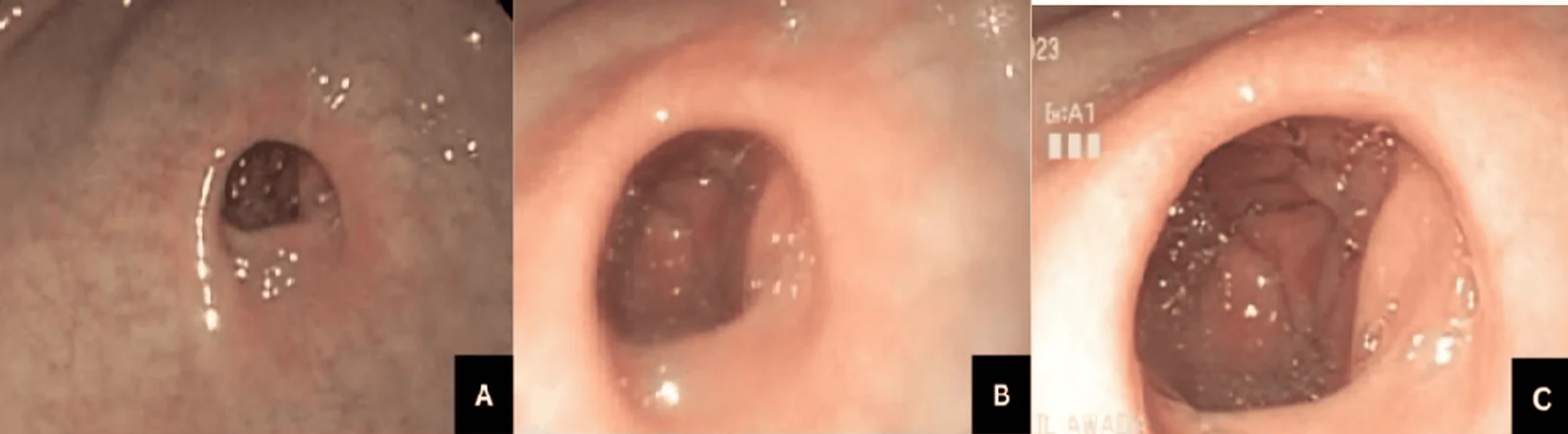
Endoscopic visualization of a gastric antral web causing progressive luminal narrowing. (A) Endoscopic view showing a small central aperture within the gastric antral web causing marked luminal narrowing. (B) Closer view demonstrating the membranous structure of the gastric web with partial obstruction of the lumen. (C) Advanced endoscopic view beyond the web, revealing the distal lumen through the narrowed opening

To further assess the degree of obstruction, an upper gastrointestinal contrast study was performed, demonstrating delayed gastric emptying with passage of contrast through a narrowed antral opening (Figure 2). Additionally, a contrast-enhanced preoperative computed tomography scan (Figure 3) revealed marked gastric distension with significant luminal dilation and relative narrowing at the distal gastric outlet, consistent with GOO. No clear extrinsic compressive mass was identified, supporting an intrinsic obstructive process.

**Figure 2:**
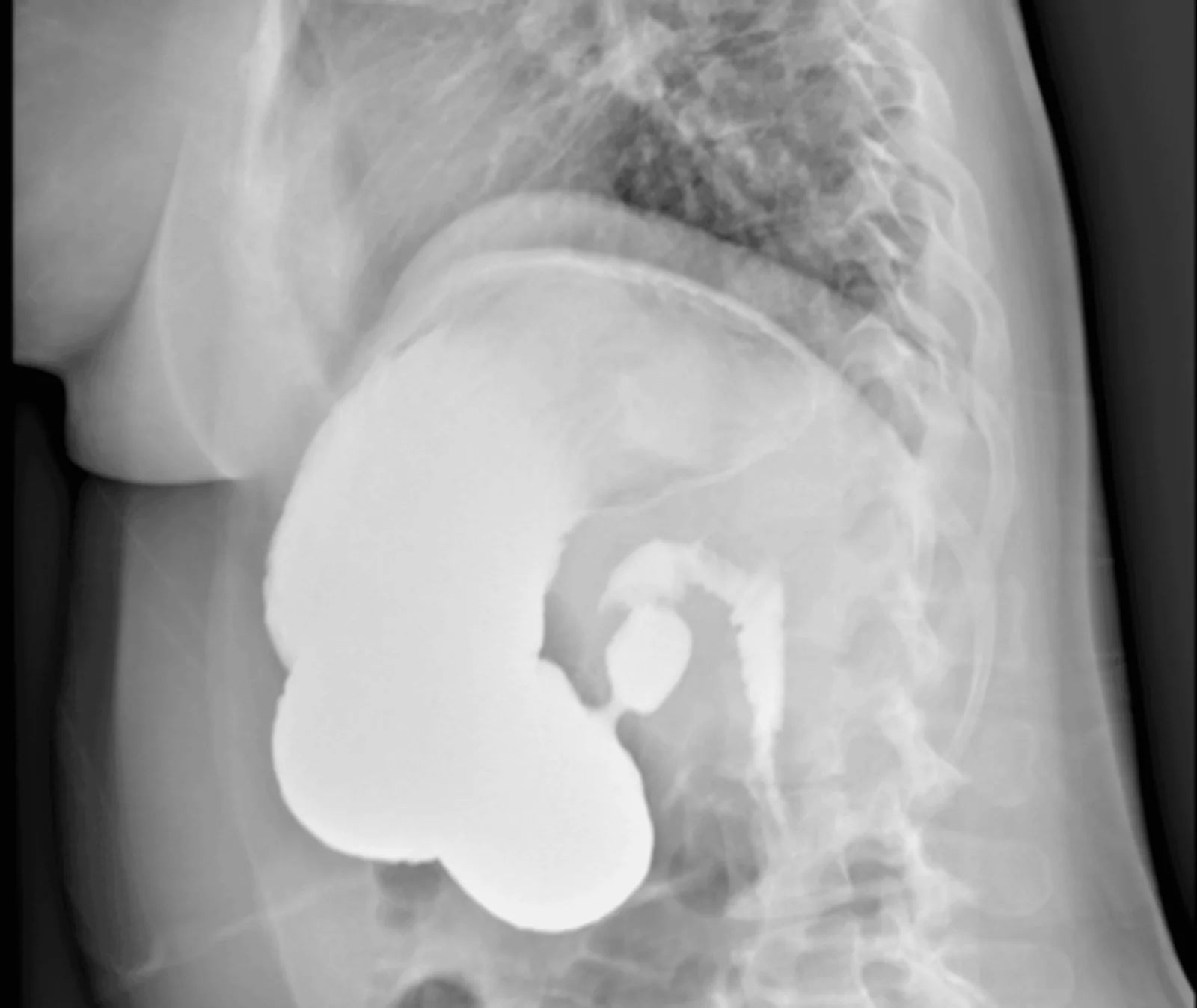
Preoperative upper gastrointestinal contrast study showing delayed gastric emptying and passage of contrast through a narrowed antral opening

**Figure 3:**
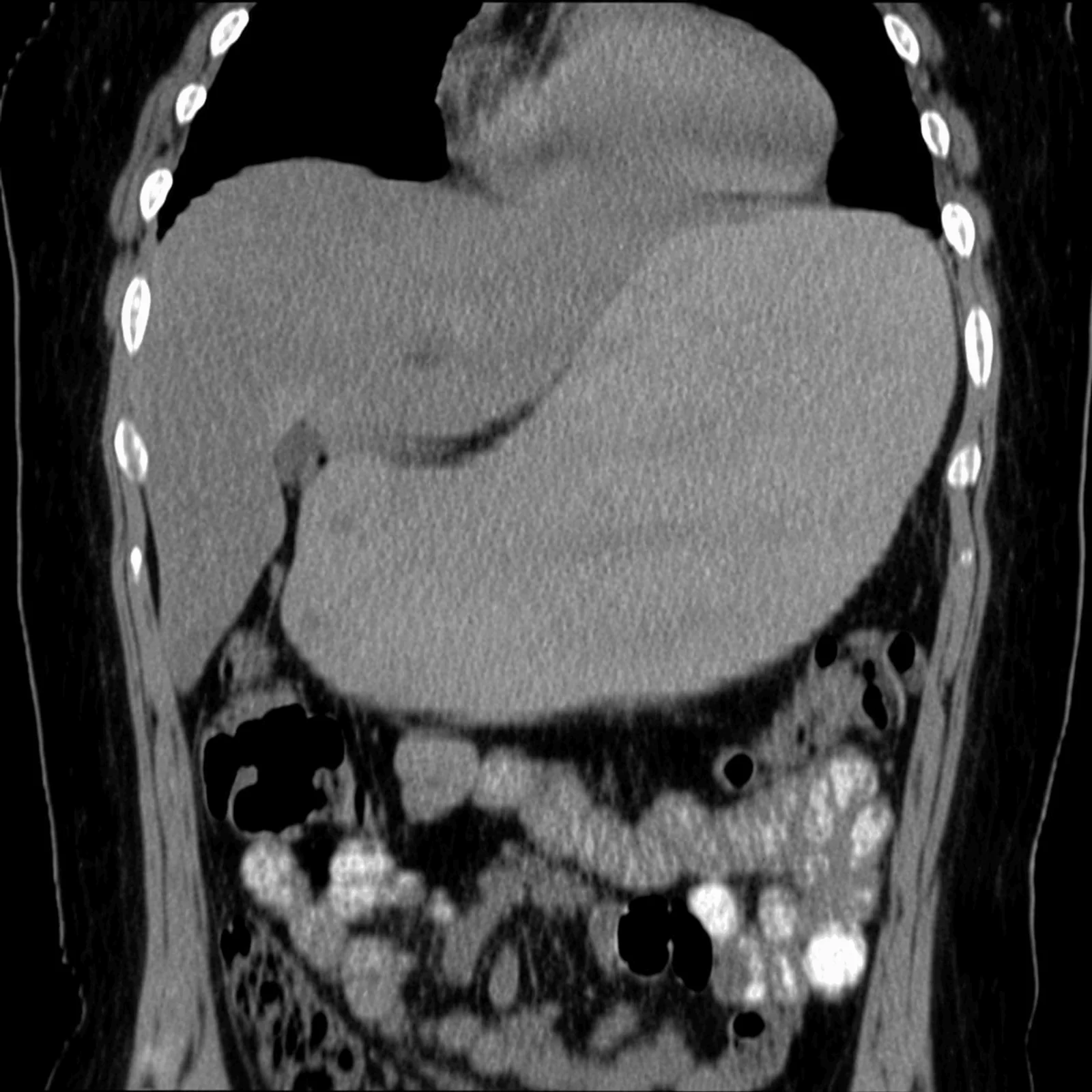
Contrast-enhanced CT scan demonstrating gastric outlet obstruction. Coronal contrast-enhanced CT image showing marked gastric distension with luminal dilation and narrowing at the level of the distal stomach/antrum, consistent with gastric outlet obstruction. No obvious external compressive lesion is observed CT: computed tomography

Following multidisciplinary discussion, surgical management was deemed the most appropriate approach given the symptomatic obstruction and anatomical findings, and given the weight of the patient and her choice of having a restrictive procedure. The patient underwent subtotal gastrectomy with gastrojejunostomy. Intraoperatively, the gastric web was confirmed, and no additional abnormalities were identified. The antral web was 3 mm thick, the aperture was 1.8 cm, and it was 3 cm distant from the pylorus.

The procedure was completed without complications. Postoperatively, the patient had an uneventful recovery, with progressive improvement in symptoms. Oral intake was gradually resumed, and the patient was maintained on proton pump inhibitors and appropriate nutritional support.

The patient was discharged on postoperative day 2 in good condition. At two-month and then at one year follow-up, she reported complete resolution of symptoms, with no recurrence of epigastric pain or postprandial fullness.

## Discussion

GOO is common in children and can be due to many causes; however, its occurrence due to GAW is a rare entity with an incidence of approximately one in 100,000 births [9]. The mechanism behind GAW in adults remains poorly understood. Some authors attribute it to an acquired process resulting from healing of pyloric ulcers, while others suggest a congenital origin due to failure of canalization following proliferation of gut luminal epithelial cells during organogenesis [10]. In our case, the mechanism of this antral web could not be determined. GAW can be classified into obstructive and nonobstructive types, with the obstructive form occurring in smaller web sizes and consequently being more symptomatic [9]. Clinical presentation depends on the size of the web and its luminal narrowing and includes emesis, abdominal fullness, pain, and decreased oral intake; in some cases, symptoms may progress to obstruction due to gradual narrowing from marginal ulcerations [7].

GAW can be detected endoscopically as a circumferential membranous structure with a central aperture through which the true pylorus may be visualized. On upper gastrointestinal series, it appears as a thin radiolucent line associated with delayed gastric emptying or, in some cases, a “double bubble” appearance [11]. Ultrasonography may also aid in diagnosis, demonstrating gastric dilatation with an echogenic diaphragmatic structure in the antrum, delayed gastric emptying, and a normal pylorus following gastric filling [12].

Management depends on symptom severity. Asymptomatic patients may be managed conservatively with dietary modifications, while symptomatic patients require intervention either endoscopically or surgically [13]. The first successful endoscopic management of GAW was reported in 1985, involving radial incisions to relieve obstruction in a pediatric patient [14]. Endoscopic techniques include balloon dilatation, needle-knife incision, and electrocautery using multiple radial cuts [15]. Patients who fail endoscopic management are candidates for surgical intervention [15]. Surgical options range from simple web excision, with or without pyloroplasty in cases of associated pyloric stenosis [9], to more extensive procedures such as distal gastrectomy in severe or refractory cases [13]. In our case, the patient underwent surgical management for the purpose of losing weight, as discussed with the surgeon, which favored this approach over endoscopic management.

## Conclusions

GAW is an uncommon condition of incomplete GOO, more often in neonatal. Awareness of this condition can lead to an earlier diagnosis. Careful clinical examination and early imaging are the key of diagnosis and prevention of complications. Both surgical treatment and endoscopic intervention are appropriate and the right choice of management should be individualized according to each case.
